# Lipid membrane interactions of self-assembling antimicrobial nanofibers: effect of PEGylation†

**DOI:** 10.1039/d0ra07679a

**Published:** 2020-09-24

**Authors:** Josefine Eilsø Nielsen, Nico König, Su Yang, Maximilian W. A. Skoda, Armando Maestro, He Dong, Marité Cárdenas, Reidar Lund

**Affiliations:** Department of Chemistry, University of Oslo 0315 Oslo Norway reidar.lund@kjemi.uio.no; Jülich Centre for Neutron Science (JCNS) and Institute for Complex Systems (ICS), Forschungszentrum Jülich GmbH 52425 Jülich Germany; Department of Chemistry & Biochemistry, The University of Texas at Arlington Arlington Texas 76019 USA; ISIS Pulsed Neutron and Muon Source, Science and Technology Facilities Council, Rutherford Appleton Laboratory Harwell Science and Innovation Campus, Didco Oxfordshire OX11 OQX UK; Institut Laue – Langevin 38000 Grenoble France; Biofilms Research Center for Biointerfaces, Department of Biomedical Science, Health and Society, Malmö University 20506 Malmö Sweden

## Abstract

Supramolecular assembly and PEGylation (attachment of a polyethylene glycol polymer chain) of peptides can be an effective strategy to develop antimicrobial peptides with increased stability, antimicrobial efficacy and hemocompatibility. However, how the self-assembly properties and PEGylation affect their lipid membrane interaction is still an unanswered question. In this work, we use state-of-the-art small angle X-ray and neutron scattering (SAXS/SANS) together with neutron reflectometry (NR) to study the membrane interaction of a series of multidomain peptides, with and without PEGylation, known to self-assemble into nanofibers. Our approach allows us to study both how the structure of the peptide and the membrane are affected by the peptide–lipid interactions. When comparing self-assembled peptides with monomeric peptides that are not able to undergo assembly due to shorter chain length, we found that the nanofibers interact more strongly with the membrane. They were found to insert into the core of the membrane as well as to absorb as intact fibres on the surface. Based on the presented results, PEGylation of the multidomain peptides leads to a slight net decrease in the membrane interaction, while the distribution of the peptide at the interface is similar to the non-PEGylated peptides. Based on the structural information, we showed that nanofibers were partially disrupted upon interaction with phospholipid membranes. This is in contrast with the considerable physical stability of the peptide in solution, which is desirable for an extended *in vivo* circulation time.

## Abstract 2

Wrane interaction of a series of self-assembling antimicrobial peptides with and without PEGylation using small angle X-ray and neutron scattering and neutron reflectometry.

## Graphical abstract

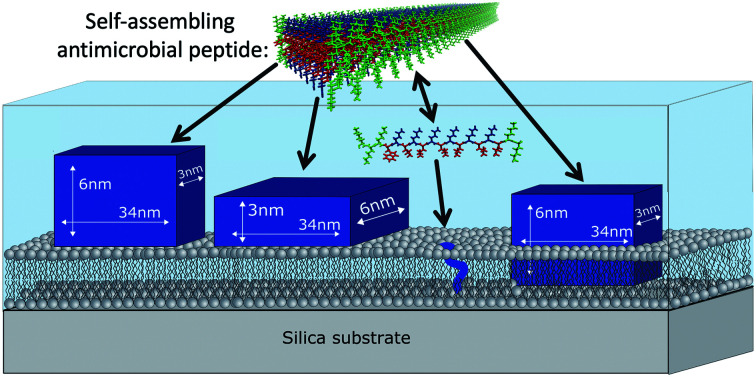

## 1. Introduction

The increase in bacterial resistance to low molecular weight antibiotics has encouraged research into the use of larger peptide or polymer-like molecules as therapeutics, which employ a different antimicrobial mechanism to overcome the existing antibiotic problem. Supramolecular assemblies of antimicrobial peptides (AMPs) have the potential to provide higher efficacy,^1–5^ decreased hemolytic response and enhanced stability to serum proteins.^1–3,5–8^ Increased activity has been reported by Beter *et al.* upon comparing self-assembled C_12_-VVAGKKKGRW-NH_2_ and KKKGRW-NH_2_ nanofibers with their corresponding soluble peptide molecules.^9^ Similar results were reported by Chang *et al.* for self-assembled cylindrical nanostructures made from C_16_–V_4_K_4_ functionalised with an (AKKARK)_2_ heparin binding Cardin-motif, which displayed strongly enhanced activity against Gram-negative bacteria above the critical micellar concentration (CMC). In the latter case it was suggested that self-assembly promotes the bacterial cytoplasmic leakage, causing blisters on disorganized membranes of Gram-negative bacteria.^10^ Contrary to the mentioned systems, Chu-Kung *et al.* found for YGAAKKAAKAAKKAAKAA (AKK) peptides, conjugated to fatty acids of varying length, that the antimicrobial activity was lost when the minimal active concentration is higher than CMC. While the conjugation of AKK with a fatty acid was shown to increase its affinity to lipid membranes, at concentrations above the CMC the self-assembled structure inhibits binding of the peptide to cell membranes.^11^ These inconsistencies indicate a required balance between hydrophobicity and assembly to optimise the antimicrobial activity, as was also reported by Molchanova and co-workers. These authors found that assembly in itself was not the cause of lowered activity for halogenated peptoids but was rather associated with increasing hydrophobicity.^12^

Cytoplasmic membrane interaction is an important feature of AMPs, either as a mechanism of action in itself, or as a step in the transmembrane transport to exert intracellular activity.^13,14^ In self-assembled peptides, the surface charge density and charge to surface area ratio differs from that of the single peptide molecules.^15^ Indeed, self-assembly has been related to both the “detergent mechanism”, where the peptides remove lipids from the membrane forming mixed micelles,^16,17^ and membrane pore-formation.^18,19^ However, the detailed effect of larger supramolecular assembly and how they structure in the presence of membranes is still an open question.

In this study we investigate the membrane interaction of a series of multidomain peptides (MDPs) previously introduced by Dong and co-workers,^20^ which exhibit antimicrobial activity against are range of different bacteria.^1^ For these MDPs the self-assembly properties have been found to directly relate to their efficacy and cytotoxicity.^1^ The MDPs are based on an ABA motif where the B group consist of a β-sheet motif of alternating hydrophilic glutamine (Q) and hydrophobic leucine (L) groups, while the A groups consist of positively charged lysine (K) residues, with the general formula K_*x*_(QL)_*y*_K_*z*_. MDP self-assembly is driven by intermolecular hydrophobic interactions and hydrogen bonding between the peptide subunits leading to a supramolecular fibrous structure.^21^ A MDP analogue used by Xu *et al.* was shown to remain stable in the presence of phospholipids, although they presented bacterial lytic abilities.^22^ Thus, it is likely that MDP membrane interaction is influenced by their self-assembly properties.

Further than affecting the antimicrobial activity and selectivity, self-assembly of AMPs affects the pharmaceutical properties of the molecules. Self-assembled antimicrobial peptides may act as a vehicle-free self-controlled delivery system, where the peptide is gradually released from the “nanoscopic depot”.^5,15,21,23,24^ This approach has the advantage of eliminating the physical encapsulation or covalent conjugation of pharmaceutical excipients in traditional formulations since it is no longer necessary to insert the active peptide in a delivery vehicle.^25^ The self-assembly approach allows for the release of active molecules without having to overcome issues related to steric hindrance or diffusion barriers.^21^ However, physical stability of the self-assembly structures under various conditions is a key parameter in the use of these systems as drug-delivery systems. König *et al.* recently showed using time resolved small angle neutron scattering (TR-SANS) that MDPs composed of K_*x*_(QL)_*y*_K_*z*_ are extremely stable at physiological relevant conditions, without any significant exchange of peptide chains in-between nanofibers over a timeframe of 2–3 days at 37 °C.^26^ This is a significant attribute for the development of long-circulation peptide-based biomaterials. However, it is yet to be determined whether the presence of a phospholipid membrane affects the physical stability of the peptides and their implication for the biological activity, which is the focus of current study.

The lack of *in vivo* stability, due to protease susceptibility, and hemocompatibility toward red blood cells remains one of the main challenges associated with using peptides in antibacterial treatment in the clinics. The K_*x*_(QL)_*y*_K_*z*_ MDPs are designed to tackle these issues both through their self-assembling nature and also due to the additional attachment of polyethylene glycol (PEG) groups to the N-terminus of the peptides. PEG improves the hemocompatibility of these peptides because it minimizes non-specific interactions with various cells, proteins and lipids in a biological environment.^6^ PEGylation has been also reported to lower the antibacterial activity in some instances depending on the length of the PEG group bound to the peptides. Singh *et al.* have shown that PEGylation of KYE28 reduces peptide binding to lipid membranes with increasing molecular weight of the PEG block, resulting in a lowered antimicrobial effect,^27^ indicating a needed balance between the reduced hemolysis and activity in the design of the peptide with regards to PEG chain length. Beyond reduction in hemolysis, PEGylation is a well-known modification of both low molecular weight drug molecules and biomacromolecules to enhance their pharmaceutical properties.^28^ For example, it's known to increase the *in vivo* half-life of parenteral drugs as well as reduce immunogenicity.^28–30^

In this work, we study the effects of MDPs with and without PEGylation on model lipid membranes using SAXS/SANS and specular neutron reflectometry (NR) at solid–liquid interfaces. NR is a powerful tool for studying peptide–membrane interactions due to the ability to resolve the detailed structure of membranes on length scales from a few Ångstrøms to tens of nanometres. NR also allows to simultaneously resolve potential lipid removal as well as peptide insertion into partly deuterated supported lipid bilayers (SLBs).^31–38^ In an earlier work, we showed that NR results can be directly compared to results from detailed modelling of small angle X-ray scattering (SAXS) data on monomeric peptide lipid bilayer using SLBs or unilamellar vesicles respectively.^31^ For supramolecular nanofibers in particular, NR has an advantage over bulk methods since it lacks 3D orientation averaging and enables precise structural determination of complex MDP–membrane structures. Here, MDPs made of K_3_W(QL)_6_K_2_ with and without PEGylation are used in combination with SLB constituted of DMPC/DMPG and studied by contrast variation NR. The results are compared to a shorter, monomeric unstructured K_3_W(QL)_3_K_2_ thereby allowing a direct comparison of the role of self-assembly on peptide–membrane interactions.

## 2. Experimental section

### 2.1 Materials and sample preparation

#### Peptide synthesis

4-Methylbenzhydrylamine (MBHA) rink amide resin, Fmoc-protected amino acids, 2-(1*H*-benzotriazol-1-yl)-1,1,3,3-tetramethyluronium hexafluorophosphate (HBTU), piperidine, diisopropylethylamine (DIPEA) and PEG2000 were purchased from Sigma-Aldrich. Dimethylformamide (DMF), acetic anhydride, trifluoroacetic acid (TFA), triisopropylsilane (TIS) and acetonitrile (ACN) were purchased from Fisher Scientific and used as received. The synthetic procedure followed the standard Fmoc-solid phase peptide synthesis method on a Prelude® peptide synthesizer. In brief, all the syntheses were set up at a 50 μmol scale using MBHA rink amide resin. The Fmoc group was deprotected utilizing 20% (v/v) piperidine/DMF for 5 minutes and repeated once. The coupling reaction was carried out for 30 min by adding 4 equivalents of Fmoc-protected amino acid, 4 equivalents of HBTU and 8 equivalents of DIPEA with respect to Fmoc-protected amino acids. After the completion of the synthesis, the N-terminus of the peptides were acetylated using DIPEA and acetic anhydride in DMF for 1 h. The completion of the coupling reaction was confirmed by the Kaiser test. The acetylated peptide was cleaved in a mixture of TFA/TIS/H_2_O (95/2.5/2.5 by volume). After 3 h, cleavage solution was filtered, and the filtrates were collected. The resins were washed three times with neat TFA and the TFA was combined with filtrate solutions and evaporated under airflow. The residual peptide solution was precipitated in cold diethyl ether, followed by centrifugation and cold diethyl ether washing for three times. The crude peptide was dried under vacuum overnight before HPLC purification. Peptides were purified using a preparative reverse phase C4 column with a linear gradient of H_2_O/ACN (5% to 95% of acetonitrile in 30 min) containing 0.05% TFA and the elution was monitored at both 230 nm and 280 nm. The HPLC fraction was collected, combined and lyophilized for 2 days. PEGylated peptide was synthesized as follows. After the final deprotection of the Fmoc group, peptide resins were treated with 4 equivalents of carboxyl terminated PEG, 4 equivalents of HBTU and 8 equivalents of DIPEA in DMF. The reaction mixture was stirred overnight. Kaiser test was performed to confirm the completion of the PEGylation reaction. The cleavage and purification steps followed the same procedure as those for acetylated peptides.

**Table:** 

Peptide	N-terminus	Peptide sequences	C-terminus
3W32	CH_3_CO–	KKKWQLQLQLKK	–CONH_2_
3W62	CH_3_CO–	KKKWQLQLQLQLQLQLKK	–CONH_2_
D–P–3W62	D–PEG2000–	KKKWQLQLQLQLQLQLKK	–CONH_2_
H–P–3W62	H–PEG2000–	KKKWQLQLQLQLQLQLKK	–CONH_2_

#### Preparation of lipid films

Synthetic DMPC (1,2-dimyristoyl-*sn-glycero*-3-phosphocholine), D54-DMPC (1,2-dimyristoyl-d54-*sn-glycero*-3-phosphocholine), DMPG (1,2-dimyristoyl-*sn-glycero*-3-phospho-(1′-*rac*-glycerol)), D54-DMPG (1,2-dimyristoyl-d54-*sn-glycero*-3-[phospho-*rac*-(1-glycerol)] (sodium salt)) and DMPE-PEG (1,2-dimyristoyl-*sn-glycero*-3-phosphoethanolamine-*N*-[methoxy(polyethylene glycol)-2000] (ammonium salt)) were purchased from Avanti Polar Lipids. Lipid films where prepared by dissolving the lipids in a methanol: chloroform solution to a 1 : 3 volume ratio, followed by solvent removal under a stream of nitrogen flow. The vials where then left under vacuum for at least one hour to ensure complete removal of organic solvents. Lipid films were then kept at −20 °C until use.

#### Matched out lipid vesicles

For the SANS and SAXS experiments the lipid films were first hydrated in a Tris buffer solution for at least one hour at 24 °C, followed by sonication in a sonication bath for 15 min, and extrusion using an Avanti mini extruder equipped with two 1 ml syringes and a 100 nm pore diameter polycarbonate filter. The lipid solution was pushed through the filter >21 times to make unilamellar lipid vesicles. For these experiments a combination of lipids with protonated and deuterated tails and D_2_O (D-) or H_2_O (H-) based 50 mM Tris buffer pH 7.4 (Sigma Aldrich) were used to match the Scattering Length Density (SLD) of both the headgroup and average lipid tail (match out vesicles). This was achieved by mixing 32 mol% d-DMPC (1,2-dimyristoyl-d54-*sn-glycero*-3-phosphocholine), 53 mol% h-DMPC, 10 mol% h-DMPG and 5 mol% DMPE-PEG in 10 mg ml^−1^ 36% D–Tris and 64% H–Tris. Addition of 5% PEGylated DMPE lipids was necessary in order to stabilise the vesicles against aggregation upon peptide addition. Provided that the lipids are randomly distributed, vesicles with this composition will essentially be contrast matched for neutrons, and thus exhibit very low scattering intensity. This enables a direct comparison of the scattering from the partly deuterated peptide D–P–3W62 in the absence or presence of lipid vesicles to detect structural changes to the peptide.

#### Supported lipid bilayers

SLBs for the NR experiments were created through fusion of tip sonicated small unilamellar vesicles (SUVs) as previously described.^39^ Prior to the experiments, the lipid films were hydrated with MilliQ water to a concentration of 0.2 mg ml^−1^ and incubated for one hour at 35 °C. The solution was then sonicated using a tip sonicator for 10 min on a 50% duty cycle (5 s on/off). The solution was mixed 1 : 1 with a 4 mM CaCl_2_ solution immediately prior to formation of lipid bilayers. The lipid suspension in CaCl_2_ was injected into the NR cell and left for approximately 10 minutes to equilibrate prior to extensive rinsing with buffer. In all the experiments, both the clean surface and the pristine lipid bilayer were fully characterized prior to peptide injection.

### 2.2 Small angle neutron scattering

SANS experiments were carried out at the time-of-flight instrument Sans2d located at the STFC ISIS Neutron and Muon Source in Didcot, United Kingdom. The sample solutions were filled into quartz cuvettes with a sample thickness of 1 mm and placed into a thermostatted sample holder rack at 37 °C. Using neutron wavelengths 2–14 Å and a detector distance of 4 m, a *Q* range of 0.004–1 Å^−1^ (*Q* = 4π sin(*θ*)/*λ* where *θ* is the scattering angle and *λ* is the neutron wavelength) was covered, with a resolution of roughly d*Q*/*Q* ≈ 2–10%. The data were reduced according to instrument standard protocols and fitted with a geometrical scattering model outlined in the ESI.†

### 2.3 Small angle X-ray scattering

The synchrotron SAXS data was collected at beamline P12 operated by EMBL Hamburg at the PETRA III storage ring (DESY, Hamburg, Germany).^40^ The data was obtained using a radiation wavelength of 1.24 Å and a detector distance of 3.0 m, covering a *Q* range of 0.0032 Å^−1^ to 0.73 Å^−1^. Data reduction was done automatically with the software available at the beam line and the 1D data were brought to absolute intensity scale using water as a primary standard.

The data were fitted with geometrical scattering models outlined in the ESI.†

### 2.4 Neutron reflectometry

NR measurements were performed using custom-made solid/liquid flow-through cells and 80 × 60 × 15 mm silicon crystals that were cleaned for 15 minutes in Piranha (3 : 1 H_2_SO_4_/H_2_O_2_) solution at 80 °C prior to the experiment. NR experiments were performed on FIGARO^41^ at Institut Laue-Langevin (Grenoble, France) and INTER at ISIS neutron source (Didcot, United Kingdom). Both instruments were used to record the time-of-flight reflectivity at two angles of incidence (Figaro: 0.8 and 3.2 degrees and Inter: 0.7 and 2.3 degrees) to cover the *Q*-range ∼0.01–0.33 Å^−1^. The instrumental resolution for Figaro was set to 
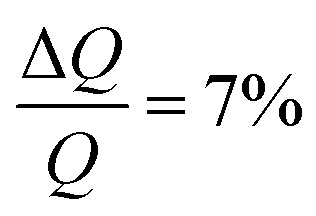
 and Inter 
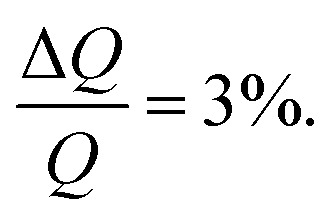
 The temperature, controlled by a circulating water bath, was maintained at 37 °C.

First, the silicon crystals were fully characterized in D_2_O and H_2_O to determine the structural parameters of the silicon oxide layer present on the surface (see ESI Fig. S1†). Second, SUVs were added and equilibrated in the cell for ∼10 min before rinsing with H–Tris. The resulting SLBs were characterized in three contrasts (D–Tris, H–Tris and a H/D–Tris mixture that matches the SLD of silicon, 62 : 38 v/v H_2_O : D_2_O, hereafter referred to as CMSi). Third, 10 ml solution (in D–Tris, CMSi and H–Tris sequentially) at the desired peptide concentration were injected into the cell at a flow rate of 1 ml min^−1^ using a syringe pump, and the resulting system was fully characterized in all three contrasts previously described. Finally, the membranes were measured again after extensive rinsing with H–Tris, CMSi and D–Tris. The use of different isotopic contrast conditions is known as the contrast variation method and it allows for simultaneous fitting of multiple reflectivity data sets, leading to reduced ambiguity and a more precise structural determination:^42^ the different contrasts highlight or suppress different parts of the system. For example, the deuterated lipid tails and deuterated PEG moieties are suppressed (or matched out) while the peptide and lipid headgroups are highlighted in D–Tris.

All reflectivity profiles were analysed using the Motofit package taking into account the experimental resolution.^43^ The NR data analysis provides information on the internal structure of thin films at an interface^44^ and, in for SLBs, this includes the composition, thickness and coverage of the different layers that compose the membrane: inner heads, lipid tails and outer heads. For fit analysis, the optical matrix method was used where the surface is modelled with three layers: one for the lipid tail and two for the hydrated head groups representing the membrane as well as solvent which were allowed to penetrate the different layers freely before peptide addition. The roughness was constrained to be the same for each interface across the whole bilayer. Upon MDP addition, the reflectivity profiles were fitted using one additional layer to account for peptide fibres absorbed on top of the bilayer (with different orientations, see sketch in Fig. 3). SLD values are calculated and fixed as given in Table S1 in the ESI.†

The error of the fit parameters for the thickness and solvent amount was determined by the Monte Carlo error analysis fitting algorithm included in the Motofit package^43^ and reflects the uncertainty of the fit. The area per molecule is calculated based on the fit parameters as
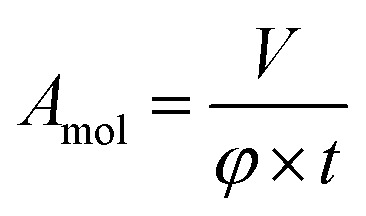
where *V* is the volume of the lipid head/tail group, *φ* is the lipid volume fraction (1-solvent [%]) and *t* is the thickness of the layer. The error in the area per molecule, *δA*_mol_, was calculated as
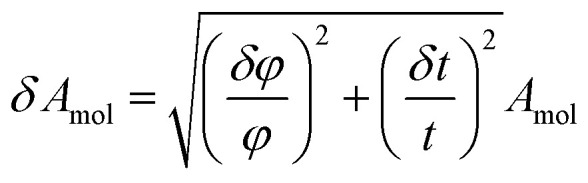


The amount of peptide inserted into the different layers of the membrane is calculated from the changes in the SLD by
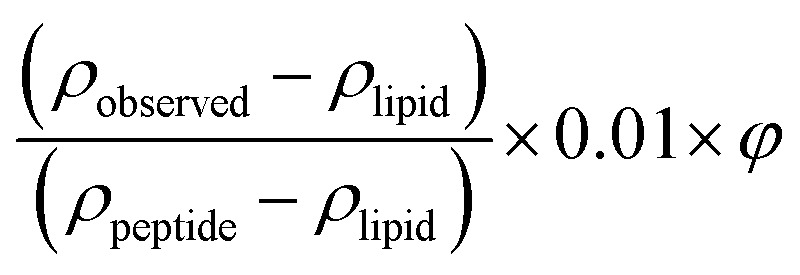
where *ρ*_observed_ is the fitted SLD of the lipid/peptide layer, and *ρ*_lipid_ and *ρ*_peptide_ is the theoretical SLD of lipid and peptide respectively.

## 3. Results and discussion

### 3.1 SANS/SAXS data confirming peptide–lipid interaction

**Fig. 1:**
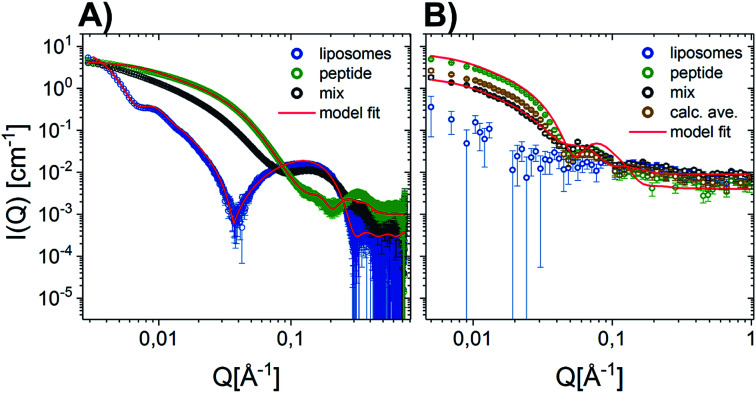
Scattering data of D–P–3W62 mixed with match-out DMPC–DMPG lipid vesicles comparing the scattering from the pure vesicles, pure peptide, mix of peptide and vesicle 9 : 10 (weight ratio) and the calculated average (average scattering from peptide and lipids measured separately). Where possible, data have been fitted with geometrical scattering models (solid lines). (A) SANS results (B) SAXS results.

Given that earlier results suggested that there were minimal interactions between MDPs and lipids,^22^ we performed a range of small-angle neutron and X-ray scattering (SANS/SAXS) studies. We aimed to qualitatively detect whether the MDPs interact with the membranes by comparing the calculated average scattering profiles for the individual components and the actual mixtures. Here, SANS enables us to focus on the peptide structure in the presence of lipids, since the lipid vesicles were matched out by the solvents and therefore do not contribute to the scattering curve (Fig. 1A). The scattering intensity for the vesicles measured by SANS alone was very low, confirming that the vesicles were properly matched out under these conditions (64% H–Tris 36% D–Tris). In contrast, the SAXS data shows a clear scattering pattern characteristic for large unilamellar vesicles, and has been fitted with an established theoretical scattering model as described in a previous publication.^45^ The neutron and X-ray scattering curves for the peptide solutions are similar to other reported results^26^ and were also fitted with a scattering model for core–shell sheet structures. The models are briefly outlined in the ESI† where the fit parameters are reported as well.

The fact that the lipid vesicles are practically matched out in the SANS experiments enables us to highlight the scattering from peptide molecules and gives an indication of how their supramolecular structure changes upon mixing with lipid vesicles. Fig. 1A demonstrates that there is a slight change in the scattering signal when comparing the peptide in the presence (“mix”) and absence of lipid vesicles (“calculated average”). This indicates an interaction between the peptides and the membrane slightly affecting the overall structure of the peptide. This is confirmed by complementary SAXS data on the exact same samples, where the scattering from the calculated average and the actual mixture differs (Fig. 1B). However, the exact peptide–lipid structures are hard to extract due to the orientational average and many components and degrees of freedom of the system. A tentative fit of the SANS data for the mixed sample, where the vesicles are practically matched out, with the scattering model used for the pure peptide yields structural parameters in good agreement with the pure peptide (compare Tables S3 in the ESI†) – with two exceptions: (1) while the free peptide in solution exhibits a uniform PEG shell of *d*_a_ ∼ *d*_b_ ∼ 30 Å thickness around the peptide fibre, the PEG distribution becomes asymmetric in the presence of lipid vesicles. The PEG layer on the longer side of the peptide core becomes compressed (*d*_a_ ∼ 13 Å) while the PEG layer on the shorter fibre side is slightly extended (*d*_b_ ∼ 35 Å). Assuming that the fibers adsorb on the surface of the vesicle, this result makes sense. (2) The apparent peptide concentration drops to ∼60% of the expected value, indicating that some peptide fibers disintegrate upon contact with the vesicles. While these findings are speculative given the structural complexity of the mixed vesicle/fibre sample, it provides additional information to the interactions. In order to investigate the membrane peptide structure, we therefore proceeded to NR.

### 3.2 Comparing the membrane interactions of shorter monomeric analogues with self-assembled peptide nanofibers

**Fig. 2:**
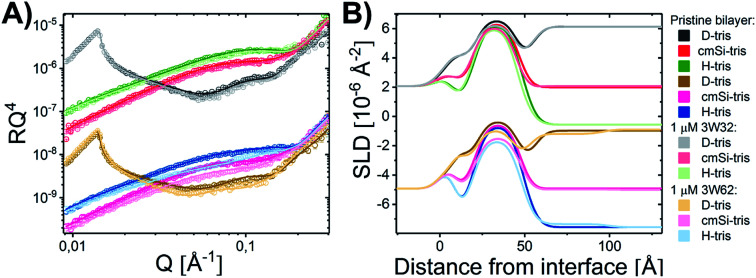
(A) NR measurements of a DMPC–DMPG (all tail deuterated) SLB at a molar ratio of 9 : 1 before and after being exposed to 1 μM 3W32 and 3W62. Reflectivity profiles for the measurements plotted together with the best fit. (B) SLD profiles resulting from the fit analysis against distance from the interface for an SLB before and after exposure to peptide. The data has been shifted in *y*-axis for clarity.

Quantitative details on the MDP–membrane interaction were instead obtained by NR. Here, we varied the peptide length, presence of PEGylation and peptide concentration systematically. First, the peptide–membrane interaction of shorter monomeric peptides (3W32) and longer self-assembling peptides with the same basic motif as 3W32 (3W62) were used. Fig. 2 shows the reflectivity profile and best fits for DMPC/DMPG bilayers at a 9 : 1 molar ratio before and after exposure to both of these peptides in H–Tris, cmSi and D–Tris contrast, together with the corresponding SLD profiles based on best fit analysis (Fig. 2B). The thickness and area per lipid calculated for the pristine bilayers (Table 1) are comparable with literature values based on MD simulations on DMPC/DMPG phospholipids^46–48^ and previous NR results.^31^

**Table 1:** Fitted parameters for tail-deuterated DMPC/DMPG membranes prior to and after exposure to 1 μM 3W32 and 3W62 peptide. The amount of peptide incorporated in the different layers is estimated based on the change in SLD observed after exposure to the peptide

Layer	*d* [Å]	Coverage [%]	SLD [10^−6^ Å^−2^]	Peptide vol%	*d* [Å]	Coverage [%]	SLD [10^−6^ Å^−2^]	Peptide vol%
**Pristine SLB**								
Water	3	0	—	—	4 ± 1	0	—	—
Head (inner)	6 ± 1	85 ± 3	1.83	—	6 ± 1	83 ± 3	1.83	—
Tail	26 ± 1	95 ± 1	6.7	—	27 ± 1	94 ± 1	6.7	—
Head (upper)	6 ± 1	85 ± 3	1.83	—	6 ± 1	83 ± 3	1.83	—
Total membrane thickness (Å)	**38 ± 2**	***A***_**mol**_ **= 63 ± 3 Å** ^**2**^			**39 ± 2**	***A***_**mol**_ **= 61 ± 2 Å** ^**2**^		

**SLB after addition of**	**1 μM 3W32**				**1 μM 3W62**			
Water	3	0	—	—	4 ± 1	0	—	—
Head (inner)	6 ± 1	85 ± 3	1.83	—	6 ± 1	85 ± 3	1.83	—
Tail/peptide	26 ± 1	95 ± 1	6.25	5 ± 1	26 ± 1	90 ± 1	6.0	11 ± 1
Head/peptide	7 ± 1	75 ± 4	1.75	8 ± 2	6 ± 1	72 ± 4	1.78	13 ± 2
Total membrane thickness (Å)	**39 ± 2**		***A***_**mol**_ **N/A**		**38 ± 2**		***A***_**mol**_ **N/A**	
Peptide layer	—	—	—	—	46 ± 1	12 ± 1	1.5/2.2/3.2 ± 0.2^a^	100

a SLD of peptide taking into account D/H exchange, see ESI Table S1. Fixed parameters during fitting.

Addition of the shorter 3W32 peptide had only a slight effect on the membrane structure (Fig. 2). The overall bilayer thickness was unaffected (when taking into account the fit error) by peptide addition. Some peptide insertion occurs as evidenced by the fact that the SLDs of the tail layer and the outer head layer in the SLBs changed upon peptide addition. Based on the changes in SLDs and the surface coverage, the amount of inserted peptide is calculated to be 5 vol% in the tail and 8 vol% in the outer head region (Table 1). These peptides exist as free chains in monomeric form in solution (as confirmed by SAXS data presented in the ESI Fig. S3†) and probably they insert as single chains in the membrane similar to other AMPs having a random coil structure such as indolicidin.^31^ However, when comparing to indolicidin, not only is the amount of inserted 3W32 in the hydrophobic lipid region significantly lower,^31^ but 3W32 seems unable to penetrate into the inner head group of the bilayer at this concentration. This might suggest that the amount of hydrophobic leucine groups is too low to provide sufficient driving force for membrane penetration. This is also reflected in the lack of assembly observed in solution, where SAXS results show that 3W32 exist as random coils rather than nanosheets as the longer 3W62 peptides (see ESI† for more information).

Contrary to 3W32, the longer peptide 3W62 had a more pronounced effect on NR data and corresponding SLD profile of the membrane for the best fits as seen from Fig. 2. Peptide addition results in a slight shift in the reflectivity curve of the D–Tris curve to lower *Q* indicating a thickening of the peptide–lipid membrane. This thickening cannot be explained by a uniform increase in the lipid membrane thickness due to peptide penetration for 3W32. Rather, addition of an uneven adsorbed peptide layer on the membrane's surface is necessary: best fits are obtained when assuming a peptide layer absorbed on top of the SLB (comparative best fits for model with and without uneven adsorbed peptise layer are shown in ESI Fig. S4†). Indeed, the SLB thickness is unaffected by peptide addition although the SLB's SLD change reveal that there is about 11% and 14% peptide insertion in the tail region and the outer head group respectively. These amounts are comparable to the inserted amounts of the shorter peptide 3W32. The additional peptide layer is 46 ± 1 Å thick with a coverage of 12 vol%.

**Fig. 3:**
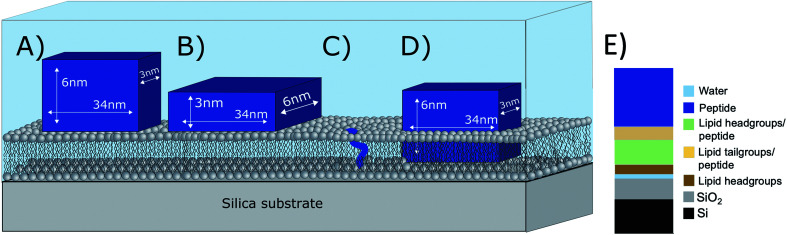
Illustration of possible positioning of the peptide nanofibers on the SLBs based on NR fit results: (A) vertical orientation (B) horizontal orientation (C) monomeric insertion. (D) Embedded orientation. The peptide nanofibers were found to have the following cross-section 26 × 58 Å^2^ with an estimated length ≥500 Å. For simplicity, the drawings are out-of-scale with respect to the long axis (peptide length). (E) Illustration on how the model used to analyse reflectivity data in Fig. 2B).

What is the origin of the extra layer on top of the SLB? As determined by SAXS, the dimensions of the peptide nanofibers are found to have an approximate cross-section of 26 × 58 Å^2^ and a length of ≥500 Å with some dispersity (see Fig. S2 and Table S3 of the ESI†). Thus, we can imagine the nanofiber as a thin and long cuboid. Taking into account the structure of the peptide^26^ with the lysine residues located at the short end of the fibres, an orientation with the nanofiber cuboid standing on its thin side on the SLB should facilitate the favourable electrostatic interaction between the positively charged lysine and the negatively charged DMPG headgroups on the surface of the SLB (as illustrated in Fig. 3A and hereby renamed to “vertical orientation”). However, the thickness of the peptide layer determined by fit analysis of NR data was 46 ± 1 Å rather than ∼61 Å. One possible explanation is that the peptide sheets are randomly placed on both the “thin” (Fig. 3A), and “thick” face (Fig. 3B, hereby named to “horizontal orientation”) or in a slightly tilted position. A more complex model dividing the layer into two distinct peptide layers allowing the density of each layer to vary freely is included in the ESI (Fig. S5 and Table S4†). These results give a combination of approximately 50 vol% of the adsorbed fibres positioning in the vertical orientation (∼60 Å thick layer) and 50 vol% in the horizontal orientation (∼30 Å thick layer) with the surface coverage of 15% and 7%, respectively. However, because the overall surface coverage of the absorbed bilayer is so low the resolution of the NR method used does not allow us to fully conclude on the orientation of the peptide at this low concentration. Monte Carlo error analysis (see ESI Fig. S6†) showed a significant level of correlation between the thickness of the two upper peptide layers using this model and therefor the simpler model of only one 46 ± 1 Å has been included in the manuscript.

The described peptide nanofiber adsorption on top of the SLB does not explain the changes observed in the SLD of the bilayer core and outer head layer. Rather this could be explained by a fraction of free peptides being able to penetrate into the bilayer either as smaller fragment of a fibre or as monomers (Fig. 3C). However, recent TR-SANS experiments on these nanofibers showed that no significant peptide release from the fibres occurred under similar experimental conditions^26^ or by NMR in the presence of a lipid membrane.^22^ For example, peptide exchange could take place directly between the absorbed peptide fibres on the surface and the lipid bilayer. In addition, the peptide fibres are formed due to intermolecular hydrogen bonds along the sheets and these bonds might be broken by competing hydrogen bonds with the phospholipid head groups.

An alternative scenario to explain the change in the SLB of the lipid bilayer is that some intact nanofibers penetrate into the SLB with its short axis facing down the membrane (Fig. 3D). This partly embedded position would explain the 46 ± 1 Å peptide layer observed on the surface of the membrane being thinner than the height of the peptide in the vertical orientation. In this scenario the peptide nanofibers are protruding 15 ± 5 Å from the SLB (with 7% surface coverage). The sum of the thickness of the membrane tail and outer head layer is approximately 33 Å, indicating that in the embedded position of the peptide the lysine residues on the bottom part of the peptide fibre positions in close proximity to the hydrated inner head region of the membrane but do not penetrate into them. This hypothesis concurs with results seen by negative stained TEM from a peptide with similar structure, where an intact peptide nanofibers was observed inserting into the outer membrane of *Escherichia coli* bacteria.^22^ Additionally, this scenario concurs with the extreme physical stability of these peptides in the absence^26^ and presence of a lipid.^22^ Additional experiments such as Cryo-EM, SANS or fluorescence microscopy could be useful to further support whether peptide sheet penetration into the lipid membrane takes place or not. Beyond the static measurements to determine the structural peptide–lipid interaction, time-resolved NR measurements showed that the interaction happens quite fast, certainly in less than 5 minutes, after which the structure has reached equilibrium (see ESI Fig. S7†). In summary, the analysis of our NR data suggests that the self-assembled peptides have a stronger membrane interaction than the monomeric peptide, confirming the increased antibacterial activity for the former ones seen in the past by Xu and co-workers.^1^

### 3.3 The effect of PEGylation on the peptide–membrane interaction

**Fig. 4:**
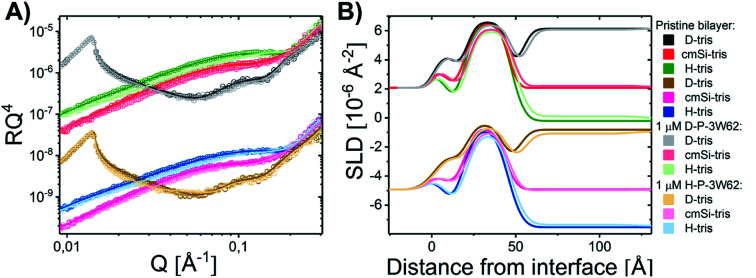
(A) NR measurements of a DMPC–DMPG SLB before and after being exposed to 1 μM H–P–3W62 and D–P–3W62. Reflectivity profiles for the measurements plotted together with the best fit. (B) SLD profiles resulting from the fit analysis against distance from the interface for an SLB before and after exposure to peptide with buffer. The data has been shifted in *y*-axis for clarity.

Earlier results by Xu and co-workers showed that MDP PEGylation does not significantly affect the antimicrobial efficacy of the resulting nanofibers.^6^ However, increased steric hindrance and solubility as well changes in hydrogen bonding in PEGylated MDPs might lead to changes in how these interact with biological membranes. To explore such effects, a PEGylated version of 3W62 was synthesized in both hydrogenated or deuterated PEG versions and are hereby named as H–P–3W62 and D–P–3W62 respectively. These peptides (1 μM) were added to pre-formed DMPC–DMPG SLB and NR data were collected (Fig. 4). The use of deuterated and hydrogenated PEGylated peptides enables more precise determination of the positioning of PEG upon peptide–membrane interaction since it provides, otherwise non-existing, contrast between the peptide and the PEG group. During data analysis, co-refinement of both the H– and D–P–3W62 systems was not possible due to small differences in the initial underlying silica surfaces and pristine bilayer structure prior to peptide addition. Across the replicates, the lipid membrane thickness of the pristine bilayers (compare Tables 1 and 2) was comparable although the surface coverage was slightly higher for one of the samples (B in Table 2 with 98% coverage while the other SLBs had 94–95% coverage).

**Table 2:** Fitted parameters for tail-deuterated DMPC/DMPG membranes prior to and after exposure to 1 μM H–P–3W62 and D–P–3W62 peptide. The amount of peptide incorporated in the different layers is estimated based on the change in SLD observed after exposure to the peptide

Layer	*d* [Å]	Coverage [%]	SLD [10^−6^ Å^−2^]	Peptide [%]	*d* [Å]	Coverage [%]	SLD [10^−6^ Å^−2^]	Peptide [%]
**Pristine SLB**								
Water	3	0	—	—	3 ± 1	0	—	—
Head (inner)	7 ± 1	82 ± 3	1.83	—	7 ± 1	84 ± 3	1.83	—
Tail	25 ± 1	94 ± 1	6.7	—	26 ± 1	98 ± 2	6.7	—
Head (upper)	7 ± 1	82 ± 3	1.83	—	7 ± 1	84 ± 3	1.83	—
Total membrane thickness (Å)	**39 ± 2**	***A***_**mol**_ **= 62 ± 3 Å** ^**2**^			**40 ± 2**	***A***_**mol**_ **= 60 ± 3 Å** ^**2**^		

**SLB after addition of**	**1 μM H–P–3W62**				**1 μM D–P–3W62**			
Water	4	100	—	—	4 ± 1	0	—	—
Head (inner)	7 ± 1	82 ± 3	1.83	—	6 ± 1	84 ± 3	1.83	—
Tail/peptide	25 ± 1	92 ± 1	6.39	6 ± 1	26 ± 1	92 ± 2	6.37	6 ± 1
Head/peptide	7 ± 1	70 ± 4	1.56	a	7 ± 1	68 ± 3	1.95	a
Total membrane thickness (Å)	**39 ± 2**		***A***_**mol**_ **N/A**		**39*s* ± 2**		***A***_**mol**_ **N/A**	
Peptide layer	64 ± 3	6 ± 1	1.1/1.4/1.9 ± 0.3		64 ± 3	6 ± 1	4.3/4.6/5.2 ± 0.2	
PEG layer	28 ± 4	2 ± 1	0.7 ± 0.3		28 ± 4	2 ± 1	7.2 ± 0.4	

a Cannot be determined with accuracy due to lack of contrast.

For both H– and D–P–3W62, only relatively small changes in the reflectivity profiles were observed (Fig. 4). However, the same model applied for the non-PEGylated peptide allowed to obtain satisfactory fits for the PEGylated peptides (Fig. 4): there was peptide adsorption on the membrane's surface, peptide insertion into the membrane as well as a slight membrane thickening. However, the extent of adsorption was lower for PEGylated peptides: the additional peptide layer was ∼64 Å thick and presented a SLD in between that of pure peptide and pure PEG. On top of this mixed peptide-PEG layer, there was an additional 28 Å layer with an SLD matching pure PEG. This suggests that the peptide nanosheets absorbed to the surface in the vertical orientation (Fig. 3A) with a highly hydrated PEG layer facing the bulk solution. The size of the PEG layer is in very good agreement with SAXS results for this peptide showing a thickness of ∼30 Å.^26^ The peptide layer's surface coverage was significantly lower than for the non-PEGylated 3W62 (12%). This might be a consequence of the increased steric hindrance and the increased peptide solubility due PEGylation making the peptide nanofibers less prone to interact with the hydrophobic part of the membrane.

Interestingly, the SLD of both the lipid core and outer lipid headgroup changed upon peptide addition (Table 2). This decrease in SLD is likely due to peptide penetration since it is unlikely for the hydrophilic PEG groups to be fully immersed into the lipid membrane core and the change in SLD was similar for both H– and D–P–3W62 (6.39 × 10^−6^ Å^−2^ or 6.37 × 10^−6^ Å^−2^, respectively). Assuming that only peptide integrates into the SLB's core, the estimated peptide insertion is 6%, and thus lower than for the non-PEGylated peptide of the same length (11%).

In contrast to the change in SLD of the SLB core region, the SLD of the outer lipid headgroup differed for H–P–3W62 (a decrease from to 1.56 × 10^−6^ Å^−2^) and D–P–3W62 (an increase to 1.95 × 10^−6^ Å^−2^). Thus, PEG inserted into the headgroup region leading to a net SLD decrease in this layer (H-PEG has a lower SLD), while the opposite is true for the deuterated PEG (with higher SLD). This suggest that the peptide inserts into the hydrophobic core of the membrane with the charged lysins positioned on the surface of the membrane partially embedded in the hydrated lipid head groups with PEG group sticking out. This suggests that the sheet nanostructures probably are destabilised and peptide insertion into the membrane probably occurs either as single chains or smaller fragments. Substantial interaction between PEG and lipid membranes with POPC and POPG lipids was reported earlier by Zhang W. and co-workers and suggested to arise from hydrogen bonding between the PEG polymer and the lipid headgroups.^49^ In summary, some peptide insertion and adsorption onto lipid membranes occurs although to a lower extent that non-PEGylated peptides, even though peptide PEGylation was reported to have no effect on the antimicrobial activity of the peptides.^6^

### 3.4 The effect of concentration on the peptide–membrane interaction

**Fig. 5:**
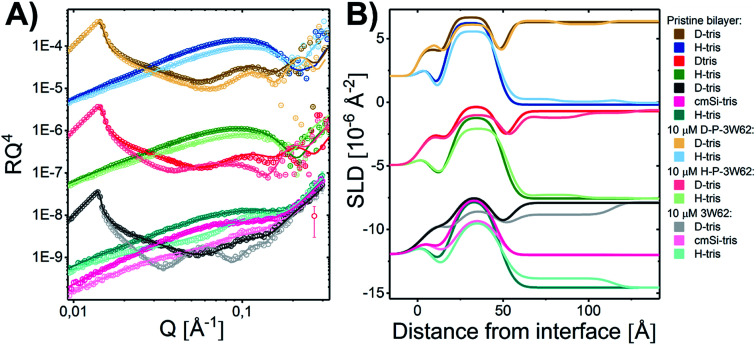
(A) NR measurements of a DMPC–DMPG SLB before and after being exposed to 10 μM 3W62 (measured at Inter beamline, ISIS, UK), H–P–3W6 and D–P–3W62 (measured at Figaro beamline at ILL, France). Reflectivity profiles for the measurements plotted together with the best fit. The differences at high *Q* for the two upper curves arise from different background subtraction at Figaro beamline at ILL as compared to Inter. (B) SLD profiles resulting from the fit analysis against distance from the interface for an SLB before and after exposure to peptide with buffer. The data have been shifted in *y*-axis for clarity.

To determine whether the membrane interaction for these peptides is cooperative or concentration dependent, separate experiments were performed at 10 μM. The reflectivity profiles for 3W62, H–P–3W62 and D–P–3W62 are shown in Fig. 5A. All pristine membranes were 38–40 Å thick with surface coverage ranging between 92 and 96%. The changes in the reflectivity profiles for the membranes before and after 10 μM peptide addition were substantially larger than for 1 μM. When comparing the PEGylated and non-PEGylated peptide it is obvious that the latter (Fig. 4A) induced a larger change in reflectivity for the D–Tris contrast. Based on fit analysis of the data for the non-PEGylated peptide 3W62, significant peptide adsorption on the membrane's surface occurred (as seen from the SLD profile in Fig. 5B): the peptide layer had a surface coverage of 34%. Moreover, substantial peptide insertion in the membrane occurred (9% in the inner headgroup leaflet, 12% in the core and 35% in the outer headgroup leaflet) with consequential lipid removal (the coverage of the tail region decreases from 96 to 88%). Similar concentration dependent effects were observed for other AMPs in the past.^31,33^ The surface coverage of the additional nanosheet layer of the 3W62 peptide is significantly higher when comparing with the 1 μM sample of the same peptide (35% compared to 12%). The thickness of the peptide layer of 60 Å corresponds with the peptide sheet adsorbing in the vertical orientation (as illustrated in Fig. 3A). Comparing with the lower concentration, we see that the higher concentration affects the inner head group (see Table 3) which seems to be adsorbing deeper into the membrane either as intact sheets, as fragments or as monomers.

**Table 3:** Fitted parameters for tail-deuterated DMPC/DMPG membranes prior to and after exposure to 10 μM 3W62, D–P–3W62 and D–P–3W62 peptide. The amount of peptide incorporated in the different layers is estimated based on the change in SLD observed after exposure to the peptide

Layer	*d* [Å]	Coverage [%]	SLD [10^−6^ Å^−2^]	Peptide [%]	*d* [Å]	Coverage [%]	SLD [10^−6^ Å^−2^]	Peptide [%]	*d* [Å]	Coverage [%]	SLD [10^−6^ Å^−2^]	Peptide [%]
**Pristine SLB**												
Water	3	0	—	—	2 ± 1	0	—	—	3	0	—	—
Head (inner)	6 ± 1	87 ± 3	1.83	—	6 ± 1	80 ± 3	1.83	—	6 ± 1	82 ± 3	1.83	—
Tail	26 ± 1	96 ± 1	6.7	—	27 ± 1	92 ± 2	6.7	—	28 ± 1	96 ± 1	6.7	—
Head (upper)	6 ± 1	87 ± 3	1.83	—	6 ± 1	80 ± 3	1.83	—	6 ± 1	82 ± 3	1.83	—
Total membrane thickness (Å)	**38 ± 2**	***A***_**mol**_ **= 61 ± 3 Å** ^**2**^			**39 ± 2**	***A***_**mol**_ **= 60 ± 3 Å** ^**2**^			**40** ± **2**	***A***_**mol**_ **= 60 ± 3 Å** ^**2**^		

**SLB after addition of**	**10 μM 3W62**				**10 μM H–P–3W62**				**10 μM D–P–3W62**			
Water	3	0	—	—	3 ± 1	0	—	—	3	0	—	—
Head (inner)	7 ± 1	82 ± 3	1.8	9 ± 4	6 ± 1	84 ± 3	1.83	—	6 ± 1	82 ± 3	1.81	
Tail/peptide	26 ± 1	88 ± 1	5.9	14 ± 1	26 ± 1	87 ± 2	6.38	5 ± 1	28 ± 1	90 ± 1	6.37	6 ± 1
Head/peptide	7 ± 1	79 ± 4	1.7	36 ± 2	7 ± 1	68 ± 3	1.74		7 ± 1	76 ± 4	2.3	
Total membrane thickness (Å)	**41 ± 2**		***A***_**mol**_ **N/A**		**39 ± 2**		***A***_**mol**_ **N/A**		**40** ± **2**		***A***_**mol**_ **N/A**	
First layer	60 ± 1	34 ± 1	1.5/2.2/3.2 ± 0.2		8 ± 2	5 ± 1	0.7 ± 0.4		5 ± 3	5 ± 1	7 ± 0.5	
Second layer					29 ± 4	14 ± 1	2.1 ± 0.3		29 ± 4	10 ± 1	2.1 ± 0.4	
Third layer	—	—	—		27 ± 4	4 ± 1	0.7 ± 0.3		26 ± 4	2 ± 1	7 ± 0.5	

As for the data described in Section 3.3 on the PEGylated peptides, the change in the membrane core SLD seems to be mainly due to peptide insertion and not PEG (estimated to be 5 ± 1% for H–P–3W62 and 6 ± 1% for D–P–3W62 as seen in Table 3), while a combination of PEG and peptide positions in the head region of the outer leaflet. Interestingly, the estimated amount of inserted peptide for the PEGylated peptide seems to be independent of the concentration in this range. This is opposed to the non-PEGylated peptide which exhibited a much more concentration dependent insertion. This suggests a concentration threshold above which there is no further nanosheet destabilization takes place possibly due to steric effects caused by the large PEG chain.

**Fig. 6:**
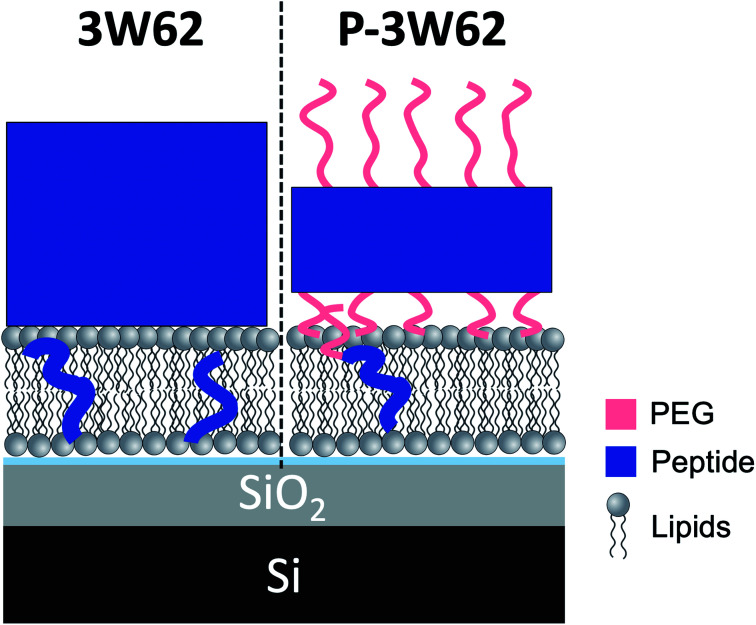
Illustration showing a comparison of the peptide position for 10 μM 3W62 and P–3W62 based on fit results of NR profiles shown in Fig. 5.

While the inserted peptide amount seems to be concentration independent, the adsorbed amount of peptide on top of the SLB increased with peptide concentration. Due to the increased amount of adsorbed nanosheets on the surface with increased peptide concentration, the independent positioning of PEG and peptide can be resolved in this case: there is a three-layer system comprised of a relative thin inner PEG layer (5–8 Å), followed by a peptide layer (29 Å) and a thicker outer PEG layer (26–27 Å) (see illustration in Fig. 6). Thus, at lower peptide concentration, similar mixed PEG/peptide layer structure should be found but cannot be resolved due to low surface concentration. This suggest a horizontal orientation positioning (as illustrated in Fig. 3B) which enables strong interaction between the PEG closest to the membrane and the lipid headgroups, leading to both partial insertion and lateral extension of PEG chains over the membrane surface. These results agree with the SANS data presented in Fig. 1 where a thinning of the PEG layer (*a*_p_) was observed when comparing data from pure peptide with data on mixed peptide–liposomes samples. Moreover, the outer PEG layer is highly hydrated and extend for 26–27 Å regardless of concentration in agreement with the dimensions found for these peptides by SAXS (hydrated PEG layer of ∼30 Å).^6,26^

## 4. Conclusions

Combining data from SANS, SAXS and NR enabled us to study the peptide–membrane interactions of MDPs, varying both the peptide's length and concentration as well as the effect of PEGylation. The results suggested that the peptide interaction is stronger for the longer peptides that self-assemble into well-defined fiber as compared to the shorter monomeric peptides. This supports the claim that self-assembled peptides have a higher antimicrobial activity. For all self-assembling peptides regardless of concentration, additional peptide layers on the surface of the SLB had to be added to fully explain the reflectivity profiles. In addition, insertion of the peptides into the core of the membrane had to be taken into account into the modelling. Addition of PEG groups to the peptide molecules seemed to decrease the peptide–membrane interaction as compared to non-PEGylated peptide. This observation does not support the retained antimicrobial activity seen in the past, indicating that the mechanism of the PEGylated peptide might not be only based on the membrane interaction. However, decreased membrane interaction would explain why the hemolytic properties decrease for the PEGylated peptides. When increasing the peptide concentration, the changes in the reflectivity profiles was more pronounced. Due to the use of peptide conjugates with both deuterated and hydrogenated PEG the spatial distribution of each component could be determined specifically using contrast variation. The PEGylated peptide molecules inserted into the membrane with only the peptide part in the lipid tail region, while a combination of peptide and PEG chains was found in the hydrated lipid headgroup region. Together the data suggested that the formation of supramolecular peptide structure increase while PEGylation decrease lipid interactions. Our results indicate that the peptide fibre structure is partly destabilized when added to phospholipid membranes, contrary to the extraordinary physical stability of the assembled peptides in the absence of lipids as previously reported.^26^ However, more specific peptide–lipid exchange studies would provide further insight into how different lipids affect the peptide structure.

## Conflicts of interest

There are no conflicts to declare.

## Supplementary Material

RA-010-D0RA07679A-s001
