# Supplementary material for: Lipid membrane interactions of self-assembling antimicrobial nanofibers: effect of PEGylation
Source: RSC Adv. 2020 Sep 24;10(58):35329–40. doi: 10.1039/d0ra07679a (PMC9056946; doi:10.1039/d0ra07679a)
Supplement: RA-010-D0RA07679A-s001 [file RA-010-D0RA07679A-s001.pdf]

Electronic Supplementary Material (ESI) for RSC Advances.
This journal is © The Royal Society of Chemistry 2020

SUPPLEMENTARY INFORMATION

Lipid Membrane Interactions of Self-assembling antimicrobial nanofibers: effect of PEGylation

Josefine Eilsø Nielsen[1], *Nico König*[1,2], Su Yang[3], *Maximilian WA Skoda*[4], *Armando Maestro*[5], *He Dong*[3], *Marité Cardenas*[6], *and Reidar Lund*[*1]

[1]*Department of Chemistry, University of Oslo, 0315 Oslo, Norway*
[2] *Jülich Centre for Neutron Science (JCNS) and Institute for Complex Systems (ICS), Forschungszentrum Jülich GmbH, 52425 Jülich, Germany*
[3]*Department of Chemistry & Biochemistry, The University of Texas at Arlington, Arlington, Texas 76019, United States*
[4]*ISIS-STFC, Rutherford Appleton Laboratory, Chilton, Oxon OX11 0QX, United Kingdom*
[5]*Institut Laue - Langevin, 38000 Grenoble, France*
[6]*Biofilms Research Center for Biointerfaces, Department of Biomedical Science, Health and Society, Malmö University, 20506 Malmö, Sweden*

**Corresponding author: reidar.lund@kjemi.uio.no*

Table S1. Theoretical SLDs for the lipids and peptide used in this study.

	Neutron SLD [10^{-6} Å^{-2}][a]
d54DMPC	
Head	1.84
Tail	6.7
d54DMPG	
Head	2.46
Tail	6.7
d54DMPC:d54DMPG 9:1	
Head	1.83
Tail	6.7
3W32	1.54[b]/2.15[c]/3.14[d]
3W62	1.54[b]/2.17[c]/3.26[d]

[a]*Calculated from the molecular component volume (based on MD simulations*[1, 2]*) and neutron scattering lengths.*
[b]*Calculated from the peptide molecular volume and the neutron scattering length.*
[c]*Calculated assuming exchange of 38% labile hydrogen atoms to deuterium in cmSi*
[d]*Calculated assuming exchange of all labile hydrogen atoms to deuterium in* D_2O

Neutron reflectivity data on pure silica crystals:

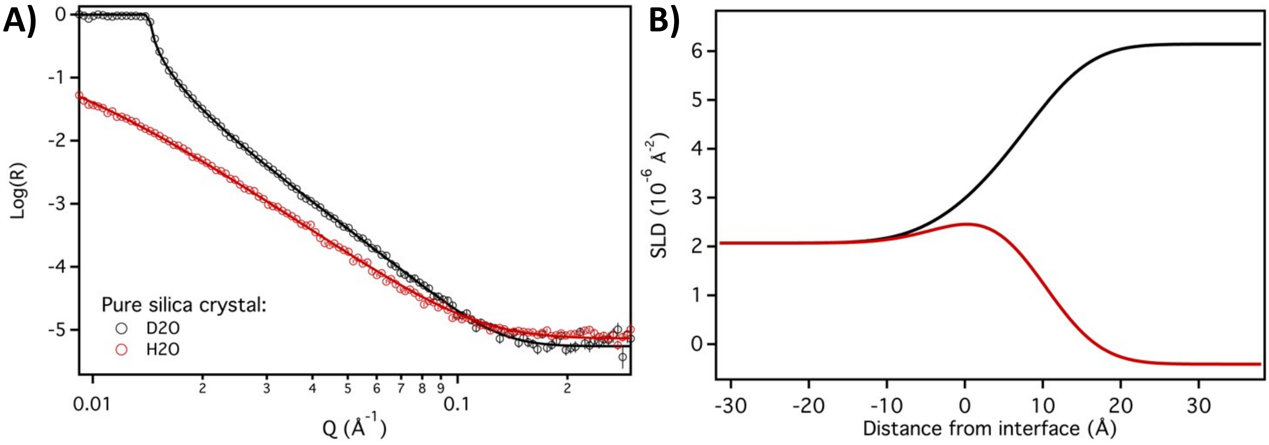

Figure S1. *A) Neutron reflectivity profiles on pure silica crystals in H_2O and D_2O plotted together with best fit. B) SLD profiles calculated from best fit of reflectivity profiles.*

Details on the small-angle scattering analysis

The SAXS data of the pure lipid vesicles shown in Figure 1A in the main manuscript were fitted with an elaborated model that is presented in detail in reference [3]. The fit parameters are given in Table S2. The SAXS and SANS data of the pure peptide solution (Figure 1 in the main manuscript) were fitted simultaneously with a core-shell-shell model that is sketched in Figure S2 and presented in detail in reference [4]. The model is an elongated prism with a rectangular cross-section. The hydrophobic tryptophan and leucine residues form the fiber core (grey) of dimensions a_i and b_i. It is surrounded by the hydrophilic peptide moieties, the backbone as well as the lysine and glutamine residues (blue), which have a thickness of a_o and b_o, respectively. Finally, PEG forms a polymer shell around the peptide fiber (pink), with thicknesses a_p and b_p, respectively. This model was also used to tentatively fit the SANS data of a peptide/vesicle mixture, where the vesicles were matched out so that the scattering originates from the peptide molecules alone. However, this model is a strong simplification and cannot account for all factors implied by the fiber-vesicle interaction, so the fit results can only serve as hints. All fit parameters are given in Table S3.

Table S2. Fit parameters of the pure vesicle SAXS data.

Parameter	Fit result
Radius	*450*
Area	*60.4**
z_{CH3}	*0**
z_{CH2o}	*14.1 ±0.2***
z_{CH2i}	*-14.0±0.2***
z_{CGo}	*16.0±0.4***
z_{CGi}	*-15.8±0.4***
Z_{HGo}	*19.5±0.2***
Z_{HGi}	*-19.3±0.2***
σ_{CH3}	*2.3**
σ_{CH2}	*4.7±0.3*
σ_{CG}	*2.1±0.2*
σ_{HG}	*4.1±0.5*
VL	*1123*
V_{CH2}	*24.8*
V_{CG}	*153**
V_{HG}	*176*
Rg PEG	*15**
dcorr	*-7*
σ_{SD}	0.22

*Hard constrained parameters are designated by * and soft constrained by limits in fitting regime indicated by **. The units for all numbers carry the appropriate power of Å.*

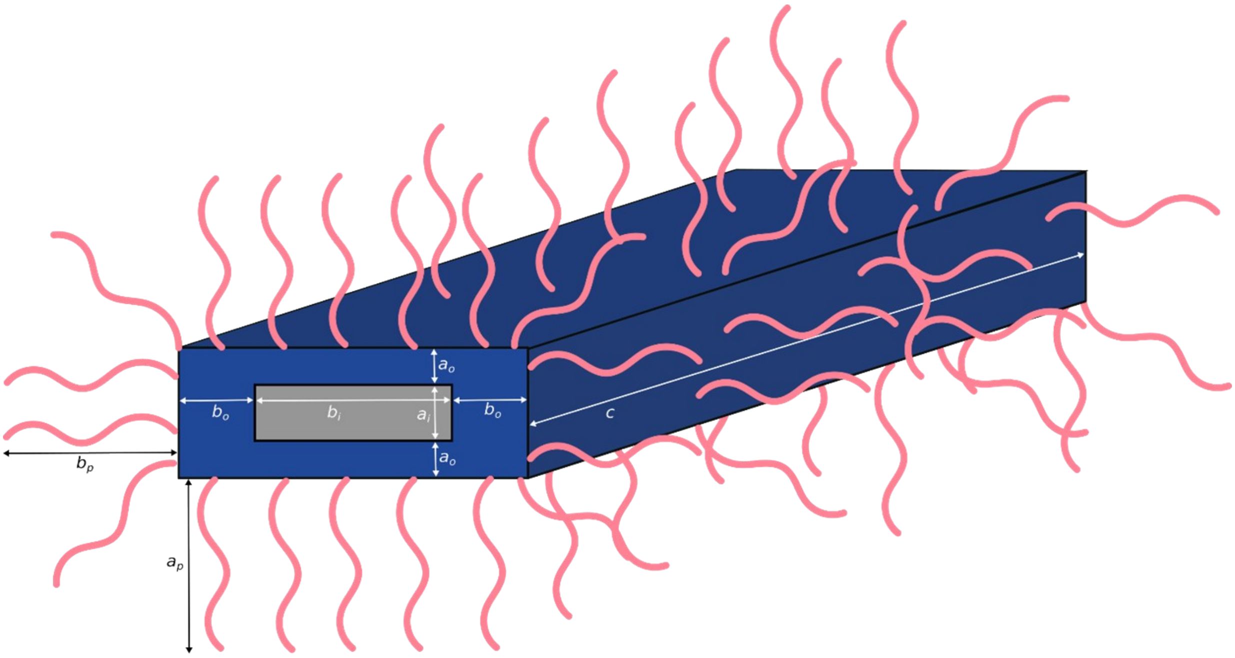

Figure S2. *Sketch of the geometrical scattering model for the peptide fibers.*

Table S3. Fit parameters of the peptide SAS data, using the model sketched in Figure S2.

	Pure peptide (SAXS)[a]	Pure peptide (SANS)[a]	Peptide in mix (SANS)
ai (Å)	9.0	9.0	8.5
ao (Å)	8.4	8.4	10.0
bi (Å)	44.3	44.3	47.7
bo (Å)	6.6	6.6	10.0
ap (Å)	29.1	29.1	12.9
bp (Å)	31.4	31.4	35.9
c (Å)	500	500	490
concentration (mg/mL)	11.0	11.0	3.0
Mpep,i (Da)	473	473	473
Mpep,o (Da)	1851	1851	1851
Mpol (Da)	2400	2400	2400
dpep,i (g/mL)	0.95	0.95	0.95
dpep,o (g/mL)	1.36	1.36	1.36
dpol (g/mL)	1.30	1.30	1.30
bpep,i (cm)	7.52E-11	-3.13E-13	-3.13E-13
bpep,o (cm)	2.79E-10	4.42E-11	4.42E-11
bpol (cm)	3.38E-10	2.22E-10	2.22E-10

[a]*Fitted simultaneously.*

Scattering data on 3W32 peptide showing a Gaussian free chain structure in solution up to concentration 10 mg/ml.

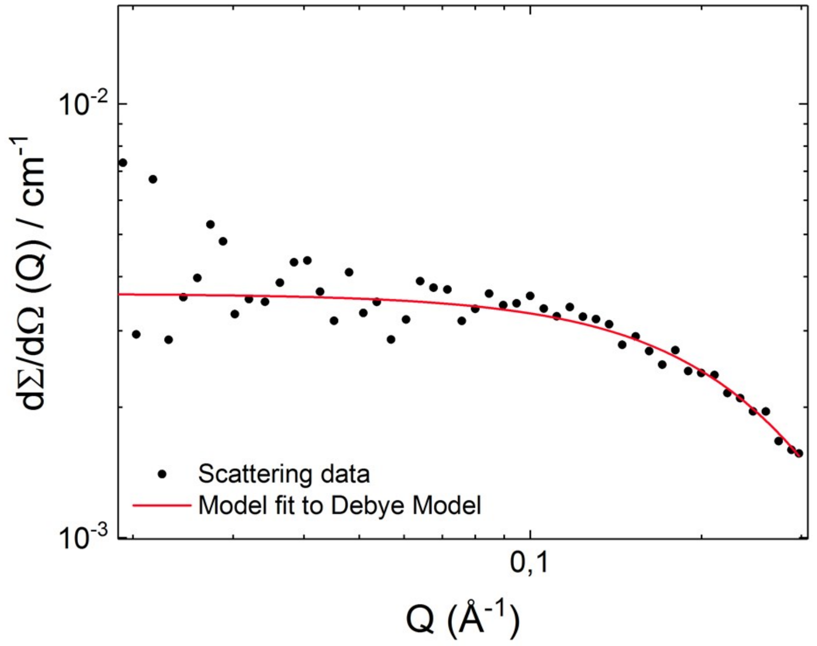

Figure S3. *SAXS data on 3W32 in solution at 5 mg/ml measured at a Bruker Nanostar lab-SAXS. Model fit using a Debye scattering model shows a Rg of 5.6 Å.*

Simulation of model with and without absorbed peptide layers on the surface of the membrane:

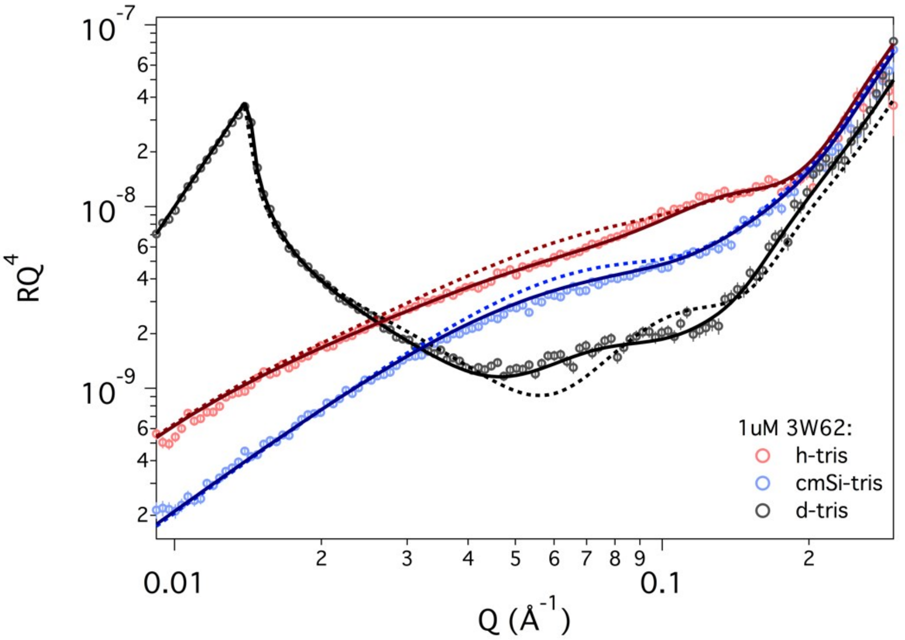

Figure S4. *Reflectivity profile for DMPC-DMPG SLB at a molar ratio of 9:1 after being exposed to 1 µM 3W62. Dotted line represents best fit using a 3 layer model with incorporation of peptide in membrane while solid line represent best fit using a 4 layer model (illustrated in Figure 3) with an additional peptide layers on the surface om the membrane. The data has been plotted as RQ^4 versus Q to better visualise the difference.*

Comparisson of 4 and 5 layer model with absorbed peptide layers on the surface of the membrane:

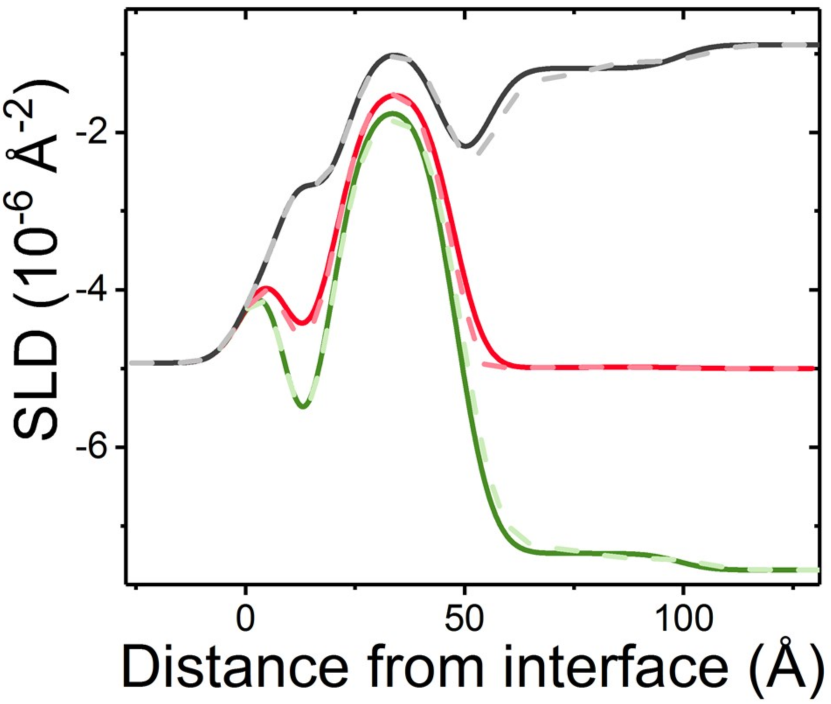

Figure S5. *SLD profile for DMPC-DMPG SLB at a molar ratio of 9:1 after being exposed to 1 µM 3W62. Solid line represents best fit using a 4 layer model with one 46 Å peptide layer on the surface of the membrane while dotted line represent best fit using a 5 layer model with two additional peptide layers of 25 and 27 Å on the surface om the membrane.*

Table S4. *Fitted parameters for tail-deuterated DMPC/DMPG membranes prior to and after exposure to 1 µM 3W62 peptide using the 5 layer model. The amount of peptide incorporated in the different layers is estimated based on the change in SLD observed after exposure to the peptide.*

Layer	d [Å]	Coverage [%]	SLD [10^{-6} Å^{-2}]	Peptide vol %
**Pristine SLB**				
Water	4 ± 1	0	-	-
Head (inner)	6± 1	83 ± 3	1.83	-
Tail	27 ± 1	94 ± 1	6.7	-
Head (upper)	6 ± 1	83 ± 3	1.83	-
Total membrane thickness (Å)	**39 ± 2**	**A_{mol} = 61 ± 2 Å^2**		
**SLB after addition of**	**1 µM 3W62**			
Water	4 ± 1	0	-	-
Head (inner)	6 ± 1	85 ± 3	1.83	-
Tail/peptide	26 ± 1	85 ± 2	6.0	11 ± 1
Head/peptide	6 ± 1	79 ± 3	1.78	14 ± 2
Total membrane thickness (Å)	**38 ± 2**		**A_{mol} N/A**	
First peptide layer	25 ± 5	15 ± 1	1.5/2.2/3.2 ± 0.2*	100
Second peptide layer	27 ± 3	8 ± 2	1.5/2.2/3.2 ± 0.2*	100

Monte Carlo error analysis on the 5 layer model for 1 μM 3W62:

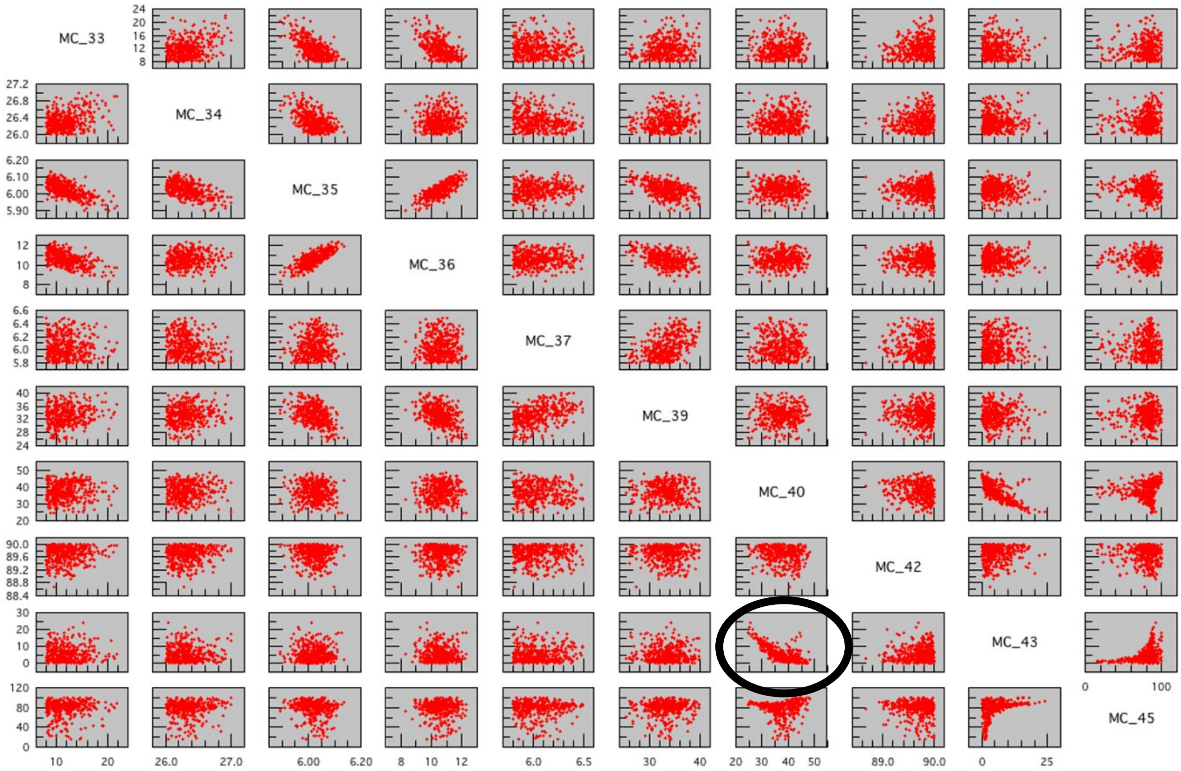

Figure S6. *Monte Carlo error analysis showing correlation between the thickness of the 4th and the 5th layer (indicated with a black circle).*

Kinetic measurements of 1 μM peptide addition:

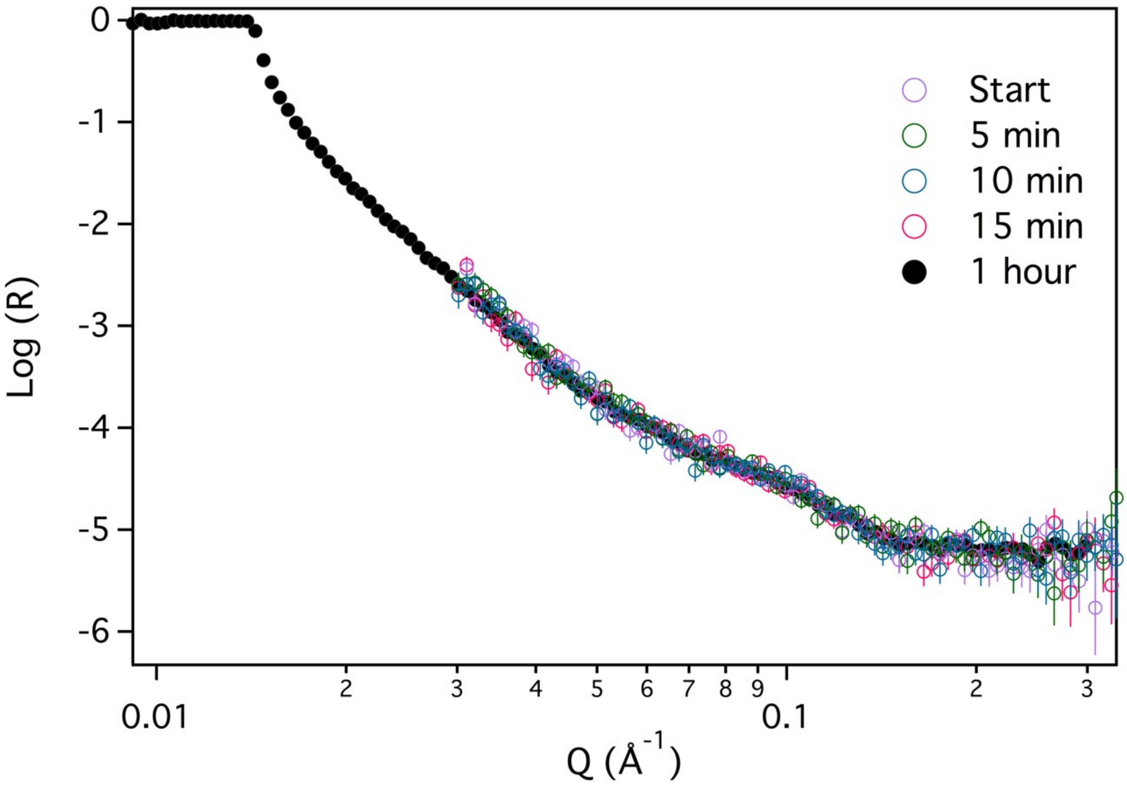

Figure S7. *Reflectivity profile for DMPC-DMPG SLB at a molar ratio of 9:1 after being exposed to 1 μM 3W62 recorded over time (only the second angle for the first 15 min). Results reveal that the peptide-lipid interaction is faster than 5 min as all the curves overlay.*

1. N. Kučerka, M.-P. Nieh and J. Katsaras, *Biochim. Biophys. Acta, Biomembr.*, 2011, **1808**, 2761-2771.
2. J. Pan, F. A. Heberle, S. Tristram-Nagle, M. Szymanski, M. Koepfinger, J. Katsaras and N. Kučerka, *Biochim. Biophys. Acta, Biomembr.*, 2012, **1818**, 2135-2148.
3. J. E. Nielsen, V. A. Bjørnestad and R. Lund, *Soft Matter*, 2018, **14**, 8750-8763.
4. N. König, J. E. Nielsen, L. Willner, A. Radulescu, N. Mahmoudi, H. Dong and R. Lund, *Extraordinary physical stability of beta-sheet nanofibers formed by self-assembly of a de novo antimicrobial peptide confirmed by small-angle scattering techniques (submitted)*, 2020.
